# Arbuscular Mycorrhiza Stimulates Biological Nitrogen Fixation in Two *Medicago* spp. through Improved Phosphorus Acquisition

**DOI:** 10.3389/fpls.2017.00390

**Published:** 2017-03-27

**Authors:** David Püschel, Martina Janoušková, Alena Voříšková, Hana Gryndlerová, Miroslav Vosátka, Jan Jansa

**Affiliations:** ^1^Department of Mycorrhizal Symbioses, Institute of Botany, Czech Academy of SciencesPrůhonice, Czechia; ^2^Laboratory of Fungal Biology, Institute of Microbiology, Czech Academy of SciencesPrague, Czechia

**Keywords:** legumes, root symbioses, rhizobia, arbuscular mycorrhiza, nitrogen acquisition, phosphorus uptake, competition, synergies

## Abstract

Legumes establish root symbioses with rhizobia that provide plants with nitrogen (N) through biological N fixation (BNF), as well as with arbuscular mycorrhizal (AM) fungi that mediate improved plant phosphorus (P) uptake. Such complex relationships complicate our understanding of nutrient acquisition by legumes and how they reward their symbiotic partners with carbon along gradients of environmental conditions. In order to disentangle the interplay between BNF and AM symbioses in two *Medicago* species (*Medicago truncatula* and *M. sativa*) along a P-fertilization gradient, we conducted a pot experiment where the rhizobia-treated plants were either inoculated or not inoculated with AM fungus *Rhizophagus irregularis* ‘PH5’ and grown in two nutrient-poor substrates subjected to one of three different P-supply levels. Throughout the experiment, all plants were fertilized with ^15^N-enriched liquid N-fertilizer to allow for assessment of BNF efficiency in terms of the fraction of N in the plants derived from the BNF (%N_BNF_). We hypothesized (1) higher %N_BNF_ coinciding with higher P supply, and (2) higher %N_BNF_ in mycorrhizal as compared to non-mycorrhizal plants under P deficiency due to mycorrhiza-mediated improvement in P nutrition. We found a strongly positive correlation between total plant P content and %N_BNF_, clearly documenting the importance of plant P nutrition for BNF efficiency. The AM symbiosis generally improved P uptake by plants and considerably stimulated the efficiency of BNF under low P availability (below 10 mg kg^-1^ water extractable P). Under high P availability (above 10 mg kg^-1^ water extractable P), the AM symbiosis brought no further benefits to the plants with respect to P nutrition even as the effects of P availability on N acquisition via BNF were further modulated by the environmental context (plant and substrate combinations). As a response to elevated P availability in the substrate, the extent of root length colonization by AM fungi was reduced, the turning points occurring at about 8 and 10 mg kg^-1^ water extractable P for *M. sativa* and *M. truncatula*, respectively. Our results indicated competition for limited C resource between the two kinds of microsymbionts and thus degradation of AM symbiotic functioning under ample P supply.

## Introduction

Legumes form two different types of root symbioses with soil microorganisms. Rhizobial symbiosis, exclusive to legumes, is established with soil diazotrophic bacteria that induce formation of nodules in host plants’ roots. Rhizobia fix atmospheric dinitrogen (N_2_) and provide it to the plants in the ammonium form that can easily be assimilated by the plant. Biological nitrogen fixation (BNF) thus contributes significantly to the nitrogen (N) budget of legumes. Its share in total N uptake by the plants is estimated to reach as high as 65–95% (Bolger et al., 1995). Arbuscular mycorrhizal (AM) symbiosis is by far more widespread among plant taxa. This association is established between the majority of terrestrial vascular plants and AM fungi from the phylum Glomeromycota (Smith and Read, 2008). AM fungi colonize plant roots and then their hyphae radiate into the surrounding soil, creating extensive networks of mycelium reaching to soil volume up to two orders of magnitude greater than what is accessible by plants alone (Raven and Edwards, 2001) and thus well beyond the depletion zone of the roots of poorly mobile nutrients such as phosphorus (P). The pivotal role of AM symbiosis occurs in enhancing plants’ uptake of such poorly mobile nutrients as P and/or zinc (Jansa et al., 2003a, 2011; Kiers et al., 2011).

**FIGURE 1:**
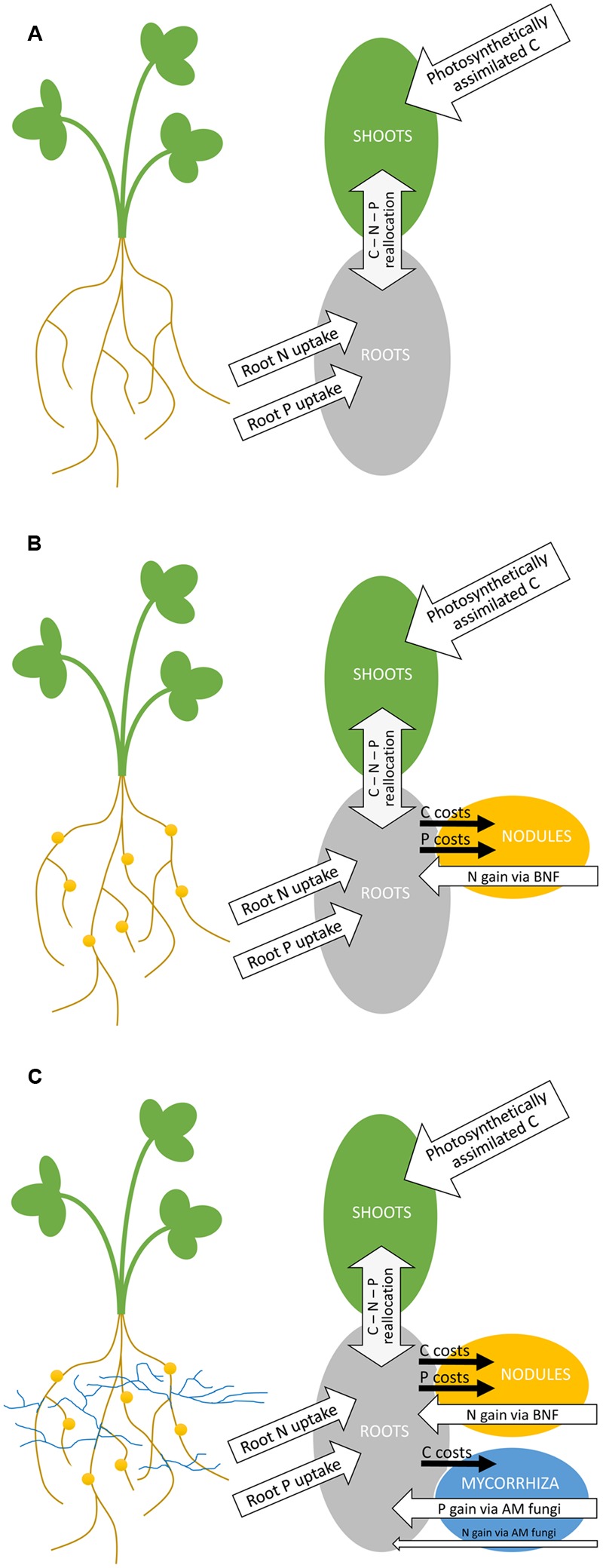
**Schematic representation of increasing complexity of carbon (C), nitrogen (N), and phosphorus (P) flows in a model leguminous plant growing either without any symbiosis (A)**, with only rhizobial symbionts mediating the biological nitrogen fixation (BNF) **(B)**, or with both rhizobial and mycorrhizal symbioses established **(C)**. Rhizobial nodules are shown as yellow circles on the roots, whereas the hyphae of arbuscular mycorrhizal (AM) fungi are represented by blue lines radiating from the roots. White arrows indicate plant acquisition pathways, gray arrows reallocation, and black arrows loss of C, N, and P, respectively.

In both rhizobial and AM symbioses, plants reward their microbial partners with photosynthetically assimilated carbon (C). The flows of C, N, and P in plants hosting both symbionts thus becomes rather complex (**Figure 1**). Each symbiosis may consume around 3–20% of recently fixed C to maintain the growth and activity and to build up energy reserves of the participating microbes (Jakobsen and Rosendahl, 1990; Kaschuk et al., 2009; Slavíková et al., 2016). The plants can partly compensate for C needs of their symbionts by increased CO_2_ assimilation (Paul and Kucey, 1981), either due to C sink stimulation or indirectly through the nutritional benefits received from the symbioses (Kaschuk et al., 2009; Řezáčová et al., 2017).

Although the functioning of either of these symbioses alone has been studied in depth through past decades, their interaction remains insufficiently explored. Synergistic effects on plants of rhizobial and mycorrhizal symbioses have been described (e.g., Kaschuk et al., 2010; Larimer et al., 2014; van der Heijden et al., 2016), but the interaction of the two symbionts may also reduce plant growth (e.g., Bethlenfalvay et al., 1982; Ballhorn et al., 2016). As pointed out in a review by Larimer et al. (2010), there is a need for more experimental studies relating the interaction of the symbionts to abiotic conditions because nutrient availability and other environmental factors may influence the outcome. For example, Saia et al. (2014) observed that AM symbiosis enhanced BNF and total plant biomass under drought stress but not under water-sufficient conditions. Ballhorn et al. (2016) reported interactive effects of AM and rhizobial symbioses depending on light availability. Surprisingly, however, there is only limited and inconclusive information on how the interaction of the two symbionts changes along the P-availability gradients (see Bethlenfalvay et al., 1982; Larimer et al., 2014).

Maintenance of rhizobial symbiosis imposes great P demand on the host plants (Jakobsen, 1985). This is because nodules have high sink-strength for P, probably due to considerable nitrogenase demand for ATP and because P concentration in microbial tissue is substantially higher than in plant cells (Jakobsen, 1985). Under low P availability to the plants, the efficiency of BNF thus often decreases (Kleinert et al., 2014). This effect is thought to be merely indirect, through intensifying P deficiency of the host plant and, as a consequence, impairing the photosynthetic capacity of the host plant, and not directly affecting nodule formation or function (Jakobsen, 1985). As AM fungi usually improve their host plants’ P status under low P availability, AM symbiosis is expected to support rhizobial activity and increase BNF. At high P availability, AM symbiosis usually does not further improve the host plant’s P budget (Smith and Read, 2008) and is therefore unlikely to increase BNF through improved P nutrition. In contrast, the host plant may become C-limited under P-sufficient conditions (Johnson et al., 1997) with the consequence that synergy of the two symbionts changes to antagonism as the two compete for C as the system-limiting resource (Bethlenfalvay et al., 1982; Reinhard et al., 1993).

To achieve a better understanding of the role of P availability in the functional interplay between rhizobial and mycorrhizal symbioses, a factorial pot experiment with two soil types and three P-fertilization levels was conducted using mycorrhizal and non-mycorrhizal individuals of the species *Medicago truncatula* and *M. sativa* inoculated with their compatible rhizobia. The plants were grown in substrates differing in pH and P availability, and fertilized with ^15^N-labeled ammonium nitrate to allow assessment of BNF contribution to the plants’ N uptake. We hypothesized that (1) the efficiency of BNF would positively correlate with P nutrition of the plants, and (2) under low P availability in the substrate, mycorrhizal plants would acquire relatively more N from BNF than would non-mycorrhizal plants due to the functional synergy between the two symbioses.

## Materials and Methods

### Experimental Design

In a greenhouse pot experiment, two model plant species, *M. truncatula* and *M. sativa*, were planted in two different substrates amended or not with mineral P-fertilizer to reach three levels of P availability for each of the substrates. All plants were inoculated with rhizobia compatible with the respective host plant species. Half of the plants were further inoculated with AM fungal isolate *Rhizophagus irregularis* ‘PH5,’ whereas the other plants grew without the AM fungus. The experiment was conducted in a fully factorial experimental design with five biological replicates per treatment, and thus it comprised 120 pots.

### Substrate and Initial P-Fertilization

A mixture (1:1, v:v) of autoclaved (at 121°C for 30 min) quartz sand (grain size < 4 mm) and autoclaved zeolite (grain size 1–2.5 mm; Zeopol s.r.o., Břeclav, Czech Republic)^1^ provided the basis of the substrate used in this study. To this sand–zeolite mixture, 10% (of final volume) of γ-irradiated (>25 kGy) soil was added. Two soils of different origins and with different physicochemical properties were used. The first soil originated from Litoměřice, Czech Republic (GPS coordinates 50.532°N, 14.110°E) and the second soil was obtained from Tänikon, Switzerland (47.489°N, 8.919°E). The two soils differed in pH and calcium content and thus were assumed to have different P saturation kinetics, effectively resulting in a 6-point P-availability gradient obtained as a combination of 2 substrates and 3 P-supply levels (see **Table 1** for selected physicochemical properties of the different substrates).

**Table 1:** Selected physico-chemical properties of sand–zeolite (1:1, v:v) substrates with 10% volumetric content of either Tänikon (Tän) or Litoměřice (LT) soil, and supplemented with either 0, 10, or 40 mg of P per pot (P0, P10, and P40, respectively).

Substrate	pH _H2O_	C [%]	N [%]	Available P [mg kg^-1^]	Total P [mg kg^-1^]
Tän P0	7.52	0.186	0.012	2.06	73.06
Tän P10	7.51	0.230	0.018	5.00	85.09
Tän P40	7.43	0.226	0.015	12.35	125.96
LT P0	8.28	0.260	0.010	3.03	108.52
LT P10	8.19	0.307	0.012	5.49	158.68
LT P40	8.33	0.214	0.008	8.32	161.81

*The pH was measured in 1:2.5 (w:v) water slurry following 30 min shaking at room temperature by pH meter FE20 (Mettler Toledo). Available (water extractable) phosphorus (P) concentrations were measured in supernatant of a water slurry (1:1, w:v, shaken for 18 h at room temperature) by malachite green method (Ohno and Zibilske, 1991). Total P concentrations were measured by the same method in acid (HNO_*3*_, 69%) extracts of the samples following incineration at 550°C for 20 h. Reported values for P concentrations are means of two separate extractions performed 9 and 19 days after application of the mineral P-fertilizer. The carbon (C) and nitrogen (N) concentrations in the samples were analyzed by Flash EA 2000 elemental analyzer (Thermo Fisher Scientific). All presented data are means of two technical replicates.*

The substrates (further referred to as “LT” or “Tän” substrate depending on the identity of the soil component) were filled into tall, 2 L plastic pots (11 cm × 11 cm × 20 cm). First, the bottom third of each pot’s volume was filled. Prior to filling the upper two thirds of the pots, the respective volume of the substrate was subjected to initial fertilization and/or mycorrhizal inoculation, as required by the specific experimental treatment (details described below).

To prevent plant growth limitation due to lack of potassium (K), magnesium (Mg), and/or calcium (Ca), these nutrients were uniformly added into all pots as initial fertilization of the substrate. The doses of 60 mg of K, 30 mg of Mg, and 30 mg of Ca (per pot) were provided by means of two separate nutrient solutions that were prepared by dissolving either 13.372 g of K_2_SO_4_ together with 30.423 g of MgSO_4_⋅7H_2_O, or 11.005 g of CaCl_2_⋅2H_2_O per 1 L of distilled water. Both solutions were applied in doses of 10 mL per pot and thoroughly mixed into the upper two thirds of the substrate filled into each pot.

The gradient of P supply comprised three levels, hereafter referred to as “P0,” “P10,” and “P40,” with either 0, 10, or 40 mg of P added to the pots (**Table 1**). This was achieved by applying one of two P solutions, prepared by dissolving either 11.563 or 46.251 g of Na_2_HPO_4_⋅12H_2_O per 1 L of distilled water. The 10 mL dose of the respective P solution or distilled water in case of P0 level was applied and mixed into the upper two thirds of the substrate filled into each pot simultaneously with the application of cations as described earlier. For P availability measured in the different substrates, please see **Table 1**.

### Mycorrhizal Inoculation

Sixty pots were inoculated with the AM fungal isolate *R. irregularis* ‘PH5.’ The isolate is maintained in the AM fungal collection of the Department of Mycorrhizal Symbioses (Institute of Botany, Czech Academy of Sciences, Průhonice, Czech Republic) in sand–zeolite–LT soil (2:2:1, v:v:v) mixture. The AM fungal inoculum cultures, established with *Zea mays* as the initial and *Desmodium* sp. as the follow-up, long-term host plant, were 16 months old when used as inoculum source. Inspection under a stereomicroscope had confirmed very abundant intraradical and extraradical sporulation of *R. irregularis*, as well as an absence of contamination by other AM fungal morphospecies. To prepare the AM fungal inoculum, the host plants’ shoots were removed and the roots were cut into ca 0.5 cm pieces and mixed back into the substrate. The material was subsequently dried at room temperature for 1 week. After thorough homogenization by mixing, the complex AM fungal inoculum (substrate+roots) was weighed into 50 g aliquots, stored temporarily in plastic bags, and then mixed into the upper two thirds of the substrate filled into each mycorrhiza-inoculated pot. This was done simultaneously with the application of cations and P (if applicable) described earlier.

To obtain an appropriate control (non-mycorrhizal, NM) treatment, a “mock” inoculum was produced in exactly the same manner as described above but using NM cultures: the same host plants were grown in the same substrate and under the same conditions as the AM fungal inoculum, but without the AM fungi. Visual inspection of the mock-inoculum cultures under a stereomicroscope confirmed the absence of AM fungal spores and/or mycelium clumps. The mock inoculum was processed and applied into the experimental pots in exactly the same manner as was the AM fungal inoculum (see above).

### Plants and Rhizobia

The seeds of *M. truncatula* J5 and *M. sativa* cv. Vlasta were surface-sterilized (10% sodium hypochlorite; 10 min) and thereafter rinsed with sterilized tap water. The plants were germinated on moist filter paper in sterilized glass Petri dishes. Those seedlings with developed cotyledon leaves were transplanted into the pots, four seedlings per pot. During transplantation, the plants were inoculated with their compatible rhizobia. *M. truncatula* was inoculated with *Sinorhizobium meliloti* strain LT10, indigenous to LT soil, which was previously selected amongst several rhizobium strains isolated from the Litoměřice field site as the most beneficial rhizobium compatible with *M. truncatula* (unpublished observation). *M. sativa* was inoculated with strain 740 (Rhizobial collection, Crop Research Institute, Prague, Czech Republic), which had been recommended for *M. sativa* plants by Lenka Kabátová (Crop Research Institute, Prague, Czech Republic, personal communication). Both of the bacterial strains were grown in TY liquid medium (Somasegaran and Hoben, 1994) on a shaker at 24°C for 3 days. The bacteria were washed with 0.5% (w:v) aqueous MgSO_4_ solution and the suspension was then adjusted to the optical density of 0.7 at 600 nm (which corresponded to approximately 2 × 10^9^ cells mL^-1^). One mL of this suspension was applied to each planting pit of individual seedlings during planting. After 1 week, the plants were thinned to two plants per pot.

Additionally, two control pots were established, one with LT and the other with Tän substrate, both of which were added with the AM fungal inoculum and fertilized with 40 mg P per pot. These pots were then planted with an isogenic mutant TRV25 (Morandi et al., 2005) of *M. truncatula* with suppressed ability to form both mycorrhizal and rhizobial symbioses. The plants were treated with the LT10 rhizobial strain as were the other experimental pots planted with *M. truncatula*. These pots were important for estimating the amount of N taken up from the substrate by P-sufficient plants in the absence of BNF. Due to spatial limitations and low availability of seeds of the mutant plant genotype, such control treatment could not have been established for each P-supply level and in a fully replicated manner.

### Plant Cultivation and ^15^N Labeling

The experiment was begun at the end of September and conducted for 9 weeks in a heated greenhouse (where temperature did not drop below 18°C at night). Natural light was supplemented with 400 W metal halide lamps set to 14 h photoperiod such that the photosynthetically active radiation flux at plant level ranged between 370 μmol⋅m^-2^⋅s^-1^ at midday and a minimum of 85 μmol⋅m^-2^⋅s^-1^ at dawn or dusk. The positions of the pots were fully randomized. The plants were watered with 25, 50, or 100 mL of distilled water per day (all pots received always the same amount of water that progressively increased with plant age).

The plants were regularly fertilized with N provided as NH_4_NO_3_ solution. N-fertilization was first applied in the 3rd week after planting (to prevent potential suppression of nodulation at early stages of plant development) and then repeated weekly, thus totaling six applications per pot. With each application, the plants were provided with 20 mg of N per pot (1.14 g of NH_4_NO_3_ was dissolved per 1 L of distilled water, and 50 mL of this solution were applied per pot).

To distinguish N uptake by plants via the root/mycorrhizal and the BNF pathways, the ammonium nitrate applied in the pots was enriched with ^15^NH_4_^15^NO_3_ (>98% ^15^N; Cambridge Isotope Laboratories, Inc., Andover, MA, USA) to reach δ ^15^N = +4491‰, corresponding to fractional abundance of ^15^N of 0.01979 (calculated value using isotopic abundance of the unlabeled and ^15^N-enriched ammonium nitrate and their molar ratio in the liquid fertilizer).

### Harvest and Sampling

The shoots were cut at the substrate surface level, pooled per pot, dried at 65°C to constant weight and weighed to obtain shoot dry weight (SDW). The compact root system with substrate was removed from the pot and the roots were shaken off to remove most of the substrate.

The roots were then carefully washed of the remaining substrate with water. Mycorrhizal colonization was assessed on roots sampled throughout the zone originally laying approximately in the 4–8 cm depth. The sampled roots were cut into ca 1 cm pieces, immersed into 10% KOH and then stained using the modified method of Koske and Gemma (1989). In brief, the roots were first macerated in 10% KOH (overnight at room temperature, then 50 min at 90°C), washed with tap water, neutralized in 2% lactic acid (20 min at 90°C), and stained with 0.05% Trypan blue in LG (lactic acid–glycerol–water, 1:1:1, v:v:v) for 30 min at 90°C plus overnight at room temperature. The next day, roots were washed with tap water and further stored in LG. Colonization was evaluated microscopically using an Olympus SZX12 dissecting microscope at 100× magnification and quantified according to the gridline intersection method (Giovannetti and Mosse, 1980) while observing at least 100 intersections per sample.

The remaining roots were also weighed fresh and then reweighed after drying at 65°C to constant weight. Root dry weight (RDW) of the entire root system per pot was then calculated. Plants’ total dry weight (TDW) was calculated as the sum of SDW and RDW. To compare plants’ growth response to inoculation in different substrate treatments, mycorrhizal growth response (MGR) of individual mycorrhizal pots was calculated from the TDW values according to the equation MGR = (M - NM_mean_)/NM_mean_ × 100% (Gange and Ayres, 1999), where M is the TDW recorded for a given mycorrhizal pot and NM_mean_ is the mean TDW of pots in the corresponding NM treatment (i.e., the same substrate and P level).

### Elemental Analyses

Prior to analyses of P and N concentrations in plant tissues, the dried samples of shoots and roots were ground to powder using a ball mill (MM200, Retsch, Haan, Germany). To determine the P concentration in plant tissues, milled samples of shoots and roots (100 mg each) were incinerated in a muffle furnace at 550°C for 12 h. The resulting ash was combined with 1 mL of concentrated (69%) HNO_3_ and briefly heated to 250°C on a hot plate. The materials was then transferred to volumetric flasks through a filter paper and brought up to 50 mL with ultrapure (18 MΩ) water. Phosphorus concentration in the extracts was then measured by colorimetry at 610 nm using a Pharmacia LKB Ultrospec III spectrophotometer by the malachite green method (Ohno and Zibilske, 1991).

The N concentrations and N isotopic composition in shoots and roots were measured using a Flash EA 2000 elemental analyzer coupled with a Delta V Advantage isotope ratio mass spectrometer (Thermo Fisher Scientific, Waltham, MA, USA).

Total N and P contents were calculated from SDW and RDW data and the concentrations of the corresponding elements in shoot and root biomass, respectively. Additionally, mycorrhizal P-uptake response (MPR) and mycorrhizal N-uptake response (MNR) were calculated from the P contents of the plants (shoots and roots combined) similarly as described above for the MGR.

### Calculation of BNF Efficiency

Assuming very similar isotopic composition of aerial N_2_ and total N in the potting substrates (fractional abundance of ^15^N in those two pools being 0.00364 and 0.00368, respectively, with the latter being the grand mean of 12 measurements of the two potting substrates amended with different levels of P before the experiment), the fraction of plant N derived from the ^15^N-labeled fertilizer (Ndff) was calculated separately for the shoots (Ndff_S_) and the roots (Ndff_R_) as follows:

(1) NdffS(mgN) = shoot N content(mg)*(15N-AT%S - 0.368)/(1.979 - 0.368)

(2) NdffR(mgN) = root N content(mg)*(15N-AT%R - 0.368)/(1.979 - 0.368),

where ^15^N-AT%_S_ and ^15^N-AT%_R_ represent isotopic composition of N in the shoots and roots expressed as ^15^N atom percent, respectively, and were measured by isotope ratio mass spectrometry.

From these values, efficiency of the BNF was calculated, here defined as the fraction of the plant N derived from biological N fixation (%N_BNF_) as follows:

(3) %NBNF(%of the plant N) = [(shoot N content - NdffS) + (root N content - NdffR)](shoot N content + root N content)

This calculation effectively ignored the contribution of seed N (likely to be very small due to the small seed size of the two experimental plant species) as well as the contribution of N contained in the components of the potting substrates. This simplification was necessary because we had not established replicated non-fixing controls for each and every combination of the potting substrate and P amendment to experimentally measure N acquisition from the differentially P-amended substrates by non-fixing plants. Inasmuch as we have experimental evidence that the substrate contribution to N acquisition by the plants is generally not very large, the simplifications described above are justifiable. Indeed, the measured contribution of substrate N to N uptake of the asymbiotic *M. truncatula* mutants supplied with the highest P level (and assuming this saturated their P demand) reached only 12.0 or 11.2 mg N for the LT and Tän substrates, respectively. This was only about 19 and 18% of their total N content when grown in the LT and Tän substrates, respectively, meaning the plants relied to a large extent for their N supply on N uptake from the liquid fertilizer (which was the only remaining N source for these plants lacking BNF as well as AM symbiosis). If extrapolated to our symbiotic experimental plants, N acquisition from the substrates would only cover about 12% of their N budget. The actual values were most likely even lower than that 12% of the plant N budget due to the functional BNF.

### Statistical Analyses

The data were analyzed using STATISTICA 12 (StatSoft Inc., USA). None of the presented data deviated significantly from the normal distribution and thus the data sets were not transformed for the statistical analyses. Data for two pots were removed from the subsequent statistical analyses: one pot from the NM treatment whose roots were colonized heavily by AM fungi (i.e., due to contamination) and one mycorrhizal pot with unusually high P content in the plant (more than twice the average of the treatment). Therefore, at least four biological replicates were retained per each treatment combination and five were included in most of them. The data were first subjected to general linear model analyses using the factors “plant,” “substrate,” and “AM fungal inoculation” as categorical predictors and “P addition” as continuous predictor in order to determine the contributions of individual factors and/or their interactions to explaining the variability in the data set (Supplementary Table S1). Individual parameters were analyzed by *t*-test to find differences between mycorrhizal and NM plants in every combination of substrate and fertilizer. A *t*-test also was used to analyze general differences in mycorrhizal colonization, MGR, MPR, and MNR between *M. truncatula* and *M. sativa* plants. The differences in mycorrhizal colonization, MGR, and MPR between P-fertilization treatments were analyzed with ANOVA followed by Tukey’s HSD test for separating the means. Correlation analyses between plant P nutrition and %N_BNF_ were carried out using a linear regression model. The slopes of regression lines for mycorrhizal and non-mycorrhizal plants were further compared in Statgraphics Plus 3.1 (Statistical Graphics Corp., USA).

## Results

### Development of the Symbiotic Microorganisms

**FIGURE 2:**
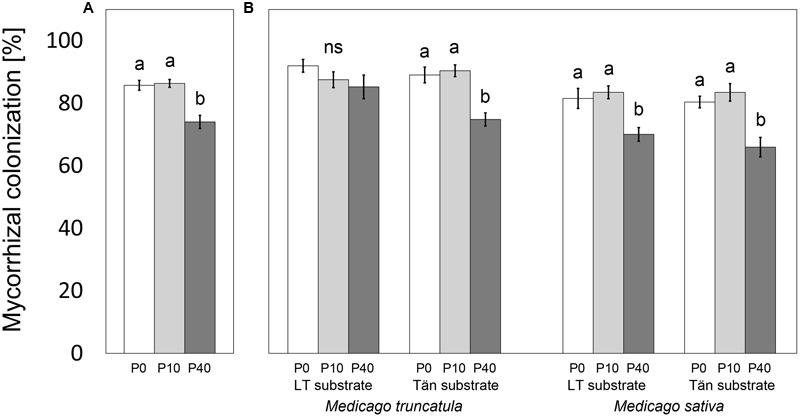
**Mycorrhizal colonization of plants inoculated with *Rhizophagus irregularis* ‘PH5’ affected by the addition of 0, 10, or 40 mg pot^-1^ of phosphorus (P0, P10, and P40, respectively).** The results are shown either for the whole data set **(A)**, or separately for *Medicago truncatula* or *M. sativa* plants grown in sand–zeolite substrate with 10% of either Litoměřice or Tänikon soil (LT or Tän substrate, respectively) **(B)**. Colonization is expressed as percentage of root length occupied by mycorrhizal fungal structures such as hyphae, arbuscules, and/or vesicles. Means ± standard errors are shown (*n* = 19 or 20 for **A**, *n* = 4 or 5 for **B**). Different letters indicate significant differences between different P levels within each plant–substrate combination according to Tukey’s HSD test (*p* < 0.05); ns, *p* ≥ 0.05. The roots of control non-mycorrhizal plants remained free of mycorrhizal fungal structures (data not shown).

All plants, excluding the two pots planted with the TRV25 mutant of *M. truncatula*, had nodules well developed in their root systems. The roots of all mycorrhizal plants were also highly (>65% of the root length) colonized with AM fungi (**Figure 2**), whereas those of NM and the mutant plants remained free of AM fungal colonization (data not shown). Mycorrhizal *M. truncatula* plants had their roots colonized to a significantly greater extent than did the *M. sativa* plants (87% vs. 77% of root length, respectively; *t*-test, *p* < 0.0001). The levels of mycorrhizal colonization were generally significantly lower in the P40 treatment compared to the less-fertilized treatments (**Figures 2A,B**), the exception being for *M. truncatula* in LT substrate (**Figure 2B**).

### Plant Growth and Mycorrhizal P Uptake

**FIGURE 3:**
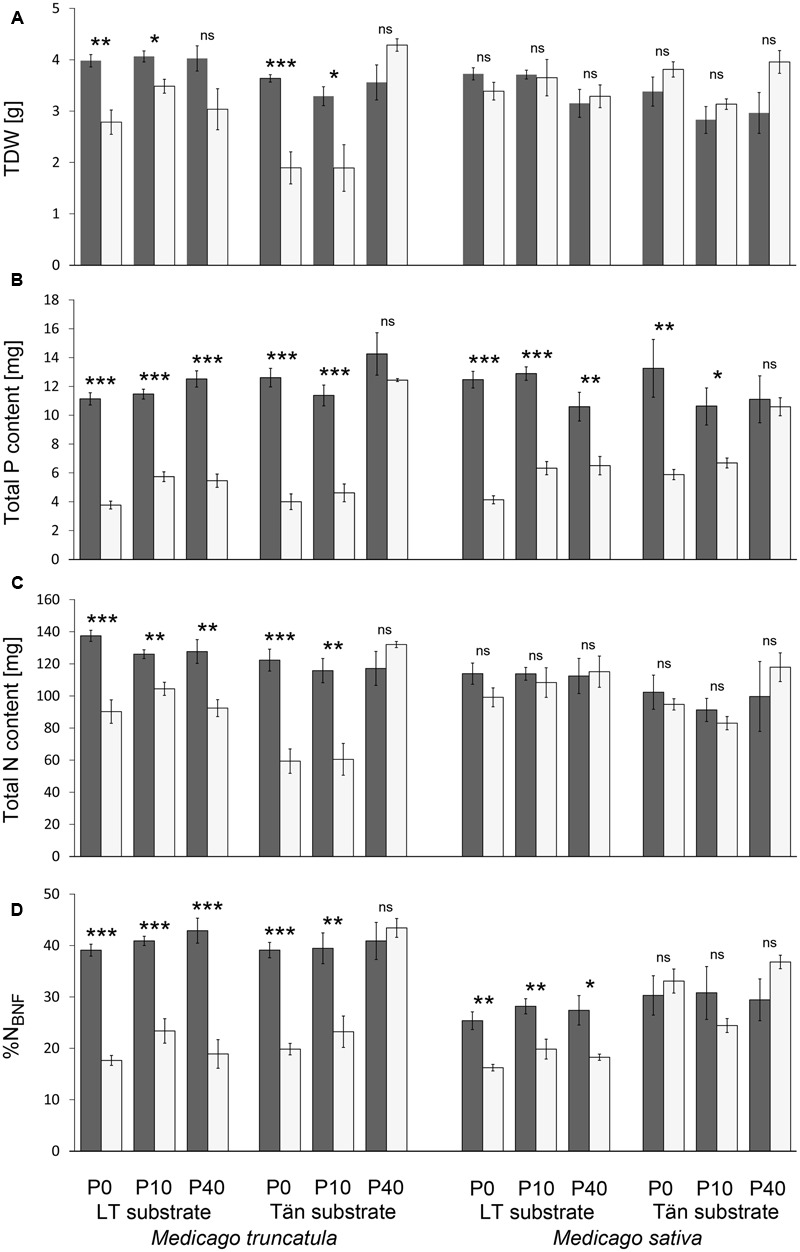
**Total plant dry weight (TDW) (A)**, total phosphorus (P) content **(B)**, total nitrogen (N) content **(C)**, and fraction of plant N derived from biological N fixation (%N_BNF_) **(D)** of mycorrhizal (dark columns) or non-mycorrhizal (light columns) *M. truncatula* or *M. sativa* plants grown in sand–zeolite substrate with 10% of either Litoměřice or Tänikon soil (LT or Tän substrate, respectively). The substrate was amended with 0, 10, or 40 mg pot^-1^ of phosphorus (P0, P10, and P40, respectively). Bars represent means ± standard errors (*n* = 4 or 5). Asterisks indicate significant differences between mycorrhizal and non-mycorrhizal plants according to *t*-test (^∗^0.01 ≤*p* < 0.05, ^∗∗^0.001 ≤*p* < 0.01, ^∗∗∗^*p* < 0.001; ns, *p* ≥ 0.05).

**FIGURE 4:**
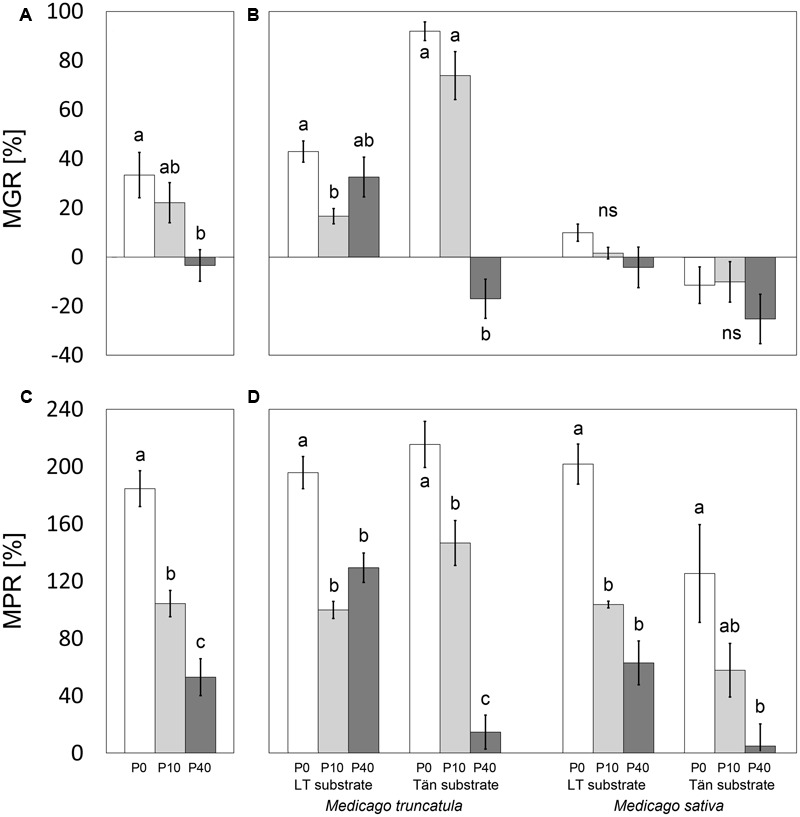
**Mycorrhizal growth response (MGR) and mycorrhizal phosphorus-uptake response (MPR) of plants along a phosphorus fertilization gradient consisting of three input levels (0, 10, and 40 mg P added per pot; P0, P10, and P40, respectively).** The results are shown either for the whole data set **(A,C)**, or separately for *M. truncatula* or *M. sativa* plants grown in sand–zeolite substrate with 10% of either Litoměřice or Tänikon soil (LT or Tän substrate, respectively). Means ± standard errors are shown (*n* = 19 or 20 for **A** and **C**, *n* = 4 or 5 for **B** and **D**). Different letters indicate significant differences between different P levels within each plant–substrate combination according to Tukey’s HSD test (*p* < 0.05); ns, *p* ≥ 0.05.

In general, the plants responded to the presence of AM fungi in terms of their biomass either positively (*M. truncatula*, except for the P40 treatment in both substrates) or else no significant effect was recorded. No case of significant negative effect of AM symbiosis on plants’ TDW was found within the individual treatments (**Figure 3A**). The calculation of MGR nevertheless did show negative values in some cases, and particularly for the P40 treatments (**Figures 4A,B**). MGR of plants, analyzed for the whole data set covering four combinations of plant species and substrates, was significantly negatively correlated with the increasing P inputs (*R*^2^ = 0.1663, *p* = 0.013). This general trend was driven, however, by a very strong correlation (*R*^2^ = 0.901, *p* < 0.0001) recorded for *M. truncatula* planted in Tän substrate, whereas correlations for the other plant–substrate combinations were not significant (data in **Figure 4B**). Highly significant differences in MGR were found between *M. truncatula* and *M. sativa* plant species, the former responding much more strongly to mycorrhiza than the later (*t*-test, *p* < 0.0001).

Mycorrhizal symbiosis significantly increased P uptake by both *Medicago* species in all substrate and fertilization treatments, except for the P40 treatment in Tän substrate (**Figure 3B**). MPR was significantly higher in *M. truncatula* plants than in *M. sativa* plants (*t*-test, *p* = 0.0413). A strong negative correlation (*R*^2^ = 0.4566, *p* < 0.0001) between MPR and P-fertilization was found for the whole data set (**Figure 4C**). Individual plant–substrate combinations followed this trend (**Figure 4D**). Only in the case of *M. truncatula* planted in LT substrate was the correlation not significant (data in **Figure 4D**).

### Biological Nitrogen Fixation

With the exception of the P40 treatment in Tän substrate, mycorrhizal symbiosis increased total plant N content and %N_BNF_ in all *M. truncatula* plants (**Figures 3C,D**). In *M. sativa* plants, by contrast, the presence of AM fungus had no effect whatsoever on N content (**Figure 3C**), but it increased %N_BNF_ in the LT substrate irrespective of the P input level (**Figure 3D**). Also, highly significant differences in MNR (*t*-test, *p* < 0.0001) evidenced the more important role of mycorrhiza in N acquisition by *M. truncatula* plants than by *M. sativa* plants.

**FIGURE 5:**
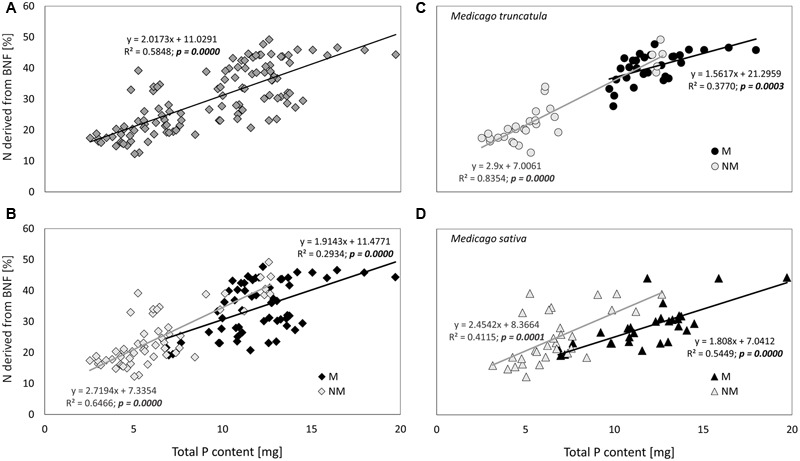
**Correlation between total phosphorus (P) content of the plants and percentage of nitrogen (N) derived from BNF.** Shown either for the whole data set **(A)**, for the data set split into mycorrhizal (M) and non-mycorrhizal (NM) plants of both species **(B)**, or further separately for *M. truncatula* **(C)** or *M. sativa* **(D)** plants. Equations along with associated *R*^2^- and *p*-values are provided for each of the linear regression models. Statistical significance (*p*-) values were derived from a goodness-of-fit test of the linear regression models.

The %N_BNF_ was strongly and positively correlated with P content in plant biomass. This was manifest not only for the data set as a whole (**Figure 5A**), but it was confirmed also when smaller data sets were tested separately (**Figures 5B–D**). While in the case of *M. truncatula* plants the slopes of regression lines for mycorrhizal and NM plants differed significantly (*p* = 0.005), and with the regression line for NM plants being steeper than that for mycorrhizal plants (**Figure 5C**), in the case of *M. sativa* plants the slopes of the regression lines for mycorrhizal and NM plants were not statistically different (**Figure 5D**). Likewise, the slopes of regression lines for mycorrhizal and NM plants pooled across the two plant species (**Figure 5B**) did not differ significantly (*p* > 0.05).

## Discussion

Using two different substrates and three levels of P supply allowed establishing a wide range of experimental conditions (P availabilities) for examining the symbiotic functioning of *Medicago* spp. along a P-fertilization gradient. The two *Medicago* species differed in their response to AM symbiosis, with *M. truncatula* being substantially more responsive to mycorrhiza formation than *M. sativa* in terms of growth, P acquisition, as well as N uptake. The two soils employed in this study as substrate components caused the P-sorption kinetics to differ between the two substrates (**Table 1**). Presumably, P was more efficiently immobilized in the calcareous LT soil with pH 7.88 (Püschel et al., 2016) than in the acidic Tän Luvisol with pH 6.2 (Jansa et al., 2003b). If the Tän substrate was fertilized with 40 mg kg^-1^ P, the water extractable P levels exceeded 10 mg kg^-1^, thereby resulting in P-sufficient conditions even for the NM plants (**Table 1** and **Figure 3**).

### Are BNF and Plant P Nutrition Related?

Considering the high P demand of the symbiotic BNF (Divito and Sadras, 2014), we had hypothesized that leguminous plants better supplied with P would, consequently, also show higher %N_BNF_. Not only was this hypothesis clearly confirmed for both *Medicago* species in association with their own compatible rhizobia, this general trend was also valid for both mycorrhizal and NM plants (**Figure 5**). These results confirmed previous observations (Ankomah et al., 1996; Vadez et al., 1999; Kuang et al., 2005) made with different leguminous plant species, although the range of environmental conditions (such as P availabilities) was usually more restricted in the previously published case studies than in our current research. Duplication of BNF efficiency due to massive P-fertilization in a mixed clover–grass sward was previously reported from a mesocosm experiment by Edwards et al. (2006). That study indirectly confirmed that the increase of BNF efficiency from 25 to 50% observed in our study due to removal of P limitation for the plants – either through AM symbiosis establishment or P-fertilization – is comparable to the effects observed under other (field-relevant) settings.

We also had expected to observe functional synergy between the two root symbionts (Barea et al., 2005), particularly if the plants were exposed to P deficiency. Our experimental evidence fully supports this second hypothesis for *M. truncatula* plants but only partly so for *M. sativa* plants. In the case of *M. truncatula*, mycorrhizal plants in all treatments with low P availability (i.e., below 10 mg kg^-1^) had significantly higher %N_BNF_ than did their respective non-mycorrhizal counterparts. This was not the same, however, for *M. sativa* plants. Although AM symbiosis still provided *M. sativa* plants with more P in exactly the same combinations of substrate and fertilization as in the case of *M. truncatula* plants, this advantage was reflected in higher %N_BNF_ in LT substrate only but not in any of the P treatments within the Tän substrate (**Figure 3**). This indicates that different functional traits of plants, the rhizobia, or the interaction between the two can respond to the outer environment in a context-specific manner. It seems, in fact, that *M. sativa* with its rhizobia particularly liked the Tän substrate, as it maintained BNF levels high in this substrate irrespective of the P-supply levels.

Under ample P supply, mycorrhizal benefits in terms of improved plant P acquisition were reduced or vanished completely (**Figures 3, 4**). This is consistent with the general consensus that mineral fertilization may render root symbionts dispensable (Morgan et al., 2005). Yet, the efficiency of BNF did not necessarily follow the same trend. Careful inspection of the regression lines plotted in **Figure 5** reveals that there were different slopes of regression lines describing how P content of *M. truncatula* related to the %N_BNF_ of the same plants (*p* = 0.005). A similar observation (though only marginally significant, with *p* = 0.088) was made also for the data set as a whole, but the slopes were not significantly different between mycorrhizal and non-mycorrhizal plants of *M. sativa* (*p* = 0.30). These results indicate that, at least in the case of *M. truncatula* (**Figure 5C**), to achieve the same BNF efficiency, mycorrhizal plants had to have substantially greater P content than did the NM plants, and the maximum %N_BNF_ values were achieved with greater difficulty or more slowly for the mycorrhizal as compared to the NM plants. These results indicate that with increasing P supply, the AM fungi and rhizobia increasingly competed for another limiting resource (at least in *M. truncatula* that also showed greater root colonization levels than did *M. sativa*). It is conceivable, based on the evidence of previous research, that the elusive limiting resource for BNF under sufficient P supply is the plant C (Morgan et al., 2005; Mortimer et al., 2009). If the metabolic energy to fix atmospheric N_2_ is in short supply due to significant mycorrhizal C sink, which could be as high as 20% of the gross photosynthetic production (Jakobsen and Rosendahl, 1990), the benefits conferred to the host by rhizobia are actually hampered by the AM fungi. Although we do not have unequivocal evidence to show that this is happening, it is highly plausible, based also on previous experimental evidence showing additivity of C costs of the two microsymbionts in tripartite root symbioses (Paul and Kucey, 1981; Mortimer et al., 2008; Millar and Ballhorn, 2013; Ballhorn et al., 2016). Under high P availability or low light conditions, the coexistence of two root symbionts becomes a burden for the plant host (Ballhorn et al., 2016). Indirect support for this theory can be observed in the suppression of root colonization by AM fungus in most of the plant–substrate treatments with increasing P-fertilization (**Figure 2**), which is consistent with the preferential allocation hypothesis (Bever, 2015).

### Relatives, Yet Functionally Different

Although using two different species of the genus *Medicago* yielded strong evidence here for a common underlying mechanism with respect to the functional interactions between mycorrhizal symbiosis and the BNF along a P-availability gradient, there were also some notable differences. One important issue that needs to be emphasized here is that both the plant and the rhizobial genotypes differed for the two plant species treatments. This was intentionally established in this manner to achieve the highest functional compatibility of the plant–bacterial partners. Thus, we were actually comparing two plant–rhizobial (biological) systems rather than two plant species *per se*.

Non-mycorrhizal *M. sativa* plants cultivated in the Tän substrate yielded surprisingly high %N_BNF_ despite that their P content under low P supply was significantly smaller than that of their mycorrhizal counterparts (**Figure 3**). It is possible that the rhizobia associated with *M. sativa* were either less P-demanding, more P-efficient, or generally more adapted to specific conditions of Tän substrate than were the bacterium used to inoculate *M. truncatula*. Such differences have been described previously and have been argued to be the result of plant–bacterial coevolution (Garau et al., 2005). Interestingly, the N contents and biomass of mycorrhizal and NM *M. sativa* plants were surprisingly similar in all substrate treatments, even though the BNF efficiency and P uptake obviously varied markedly (**Figure 3**). We therefore assume that *M. sativa* might actually better compensate for the missing symbiotic benefits through more dynamic root traits such as greater plasticity of root branching (Lynch, 2007; Nibau et al., 2008) and/or root exudation (Rao et al., 2016). Inasmuch as these traits were not recorded in our study, however, this remains a matter of speculation, although it does point to possible mechanisms accounting for why different plants vary in their response and/or dependency on mycorrhizal and other symbioses (Linderman and Davis, 2004; Jakobsen et al., 2005).

## Conclusion

Working with a large environmental (P availability) gradient established by using two different kinds of substrates in combination with three levels of mineral P inputs, we show here that AM symbiosis clearly promotes BNF efficiency, particularly in the case of low P supply. This effect was most likely mediated by improved P acquisition of the mycorrhizal as compared to the NM plants under conditions of low P. With increasing P inputs, however, the costs of the AM symbiosis (at least in the more heavily colonized *M. truncatula*) become more and more apparent, resulting in a lower P use efficiency (or in luxurious P uptake) of the mycorrhizal plants as compared to the NM plants and without concomitant increases in plant biomass production. Based upon Liebig’s law of the minimum (Johnson et al., 2015), therefore, we conclude that there was strong competition between the symbionts and the plants for another resource, thereby preventing the occurrence of a significant positive growth response in the plants at higher P-supply levels. Most likely, this competition was for carbon (Ballhorn et al., 2016). In response to sufficient (or even luxurious) P supply at higher P-fertilization levels, mycorrhizal root colonization levels were reduced. Although this was in accordance with previous reports (Treseder, 2004), this obviously was not effective enough to counteract the C drain to the AM fungus, at least not in the *M. truncatula*. Although general reduction of root colonization at higher P-fertilization levels was true for both *Medicago* species (each in association with its compatible rhizobia), notable differences were observed between the two plant species. These could be due either to the plants or the rhizobial strains used and reflect such factors as inherent tolerance of the particular rhizobia to deviation from their pH optima, different architecture of the root systems, differential efficiency of plant genotypes in mineral nutrient use and/or redistribution, root exudation patterns, or other mechanisms. Disentangling these factors would require further experimental efforts, and particularly with respect to quantifying the C costs of the two root symbioses under a range of environmental conditions.

## Author Contributions

DP, MJ, and JJ designed the experiment, which was then carried out mainly by DP. HG conducted the P and N analyses, and JJ calculated the BNF efficiency. DP conducted the statistical analyses. All authors contributed to interpreting the results. DP and JJ did most of the writing, whereas MJ, AV, and MV critically commented on earlier versions of the manuscript.

## Conflict of Interest Statement

The authors declare that the research was conducted in the absence of any commercial or financial relationships that could be construed as a potential conflict of interest.

## Footnotes

1 http://www.zeopol.com
