# Supplementary material for: Arbuscular Mycorrhiza Stimulates Biological Nitrogen Fixation in Two Medicago spp. through Improved Phosphorus Acquisition
Source: Front Plant Sci. 2017 Mar 27;8:390. doi: 10.3389/fpls.2017.00390 (PMC5366336; doi:10.3389/fpls.2017.00390)
Supplement: Supplementary file 1 [file Table_1.DOCX]

Supplementary information

**TABLE S1 |** Effects of experimental factors (plant: *Medicago truncatula* or *M. sativa*; soil: sand–zeolite substrate with 10% of LT or Tän soil; mycorrhizal inoculation: presence or absence of *Rhizophagus irregularis* ‘PH5’; phosphorus (P) addition: 0, 10 or 40 mg of P per pot) and their interactions on various plant parameters according to a general linear model (GLM) analysis using the P addition level as the continuous predictor. BNF – biological nitrogen fixation; N – nitrogen; MGR – mycorrhizal growth response, MPR – mycorrhizal P-uptake response, MNR – mycorrhizal N-uptake response. Presented are *F* and *p* values for each of the separate analyses. Significant factors or their interactions (*p* < 0.05) are highlighted in red.

|  | Total dry weight | | N derived from BNF | | Total P content | | Total N content | |
| --- | --- | --- | --- | --- | --- | --- | --- | --- |
|  | F | p | F | p | F | p | F | p |
| Plant (1) | 8.59 | 0.0042 | 6.37 | 0.0131 | 6.71 | 0.0110 | 0.01 | 0.9224 |
| Soil (2) | 19.91 | 0.0000 | 4.97 | 0.0280 | 0.01 | 0.9118 | 21.43 | 0.0000 |
| Inoculation (3) | 21.13 | 0.0000 | 75.74 | 0.0000 | 239.76 | 0.0000 | 43.17 | 0.0000 |
| Phosphorus (4) | 3.63 | 0.0597 | 14.88 | 0.0002 | 26.56 | 0.0000 | 10.61 | 0.0015 |
| 1 × 2 | 5.59 | 0.0200 | 9.12 | 0.0032 | 0.22 | 0.6375 | 2.15 | 0.1455 |
| 1 × 3 | 21.78 | 0.0000 | 25.09 | 0.0000 | 0.04 | 0.8358 | 17.00 | 0.0001 |
| 2 × 3 | 0.52 | 0.4733 | 0.92 | 0.3387 | 0.12 | 0.7275 | 2.61 | 0.1092 |
| 1 × 4 | 11.44 | 0.0010 | 4.72 | 0.0321 | 10.85 | 0.0014 | 0.49 | 0.4860 |
| 2 × 4 | 12.05 | 0.0008 | 7.82 | 0.0062 | 14.28 | 0.0003 | 9.55 | 0.0026 |
| 3 × 4 | 13.29 | 0.0004 | 7.16 | 0.0087 | 27.73 | 0.0000 | 15.92 | 0.0001 |
| 1 × 2 × 3 | 6.55 | 0.0120 | 2.12 | 0.1486 | 3.00 | 0.0863 | 2.98 | 0.0873 |
| 1 × 2 × 4 | 2.93 | 0.0899 | 4.47 | 0.0370 | 2.49 | 0.1177 | 4.47 | 0.0370 |
| 1 × 3 × 4 | 2.13 | 0.1472 | 1.26 | 0.2641 | 1.53 | 0.2188 | 1.60 | 0.2095 |
| 2 × 3 × 4 | 7.98 | 0.0057 | 13.27 | 0.0004 | 8.63 | 0.0041 | 7.69 | 0.0066 |
| 1 × 2 × 3 × 4 | 5.55 | 0.0204 | 3.96 | 0.0492 | 2.13 | 0.1471 | 4.02 | 0.0476 |
|  | Shoot P concentration | | Root P concentration | | Shoot N concentration | | Root N concentration | |
|  | F | p | F | p | F | p | F | p |
| Plant (1) | 5.94 | 0.0166 | 11.00 | 0.0013 | 18.86 | 0.0000 | 1.94 | 0.1670 |
| Soil (2) | 21.98 | 0.0000 | 40.90 | 0.0000 | 0.04 | 0.8420 | 6.38 | 0.0131 |
| Inoculation (3) | 178.76 | 0.0000 | 457.39 | 0.0000 | 2.90 | 0.0918 | 17.88 | 0.0001 |
| Phosphorus (4) | 45.42 | 0.0000 | 12.39 | 0.0006 | 8.43 | 0.0045 | 4.18 | 0.0434 |
| 1 × 2 | 10.91 | 0.0013 | 0.00 | 1.0000 | 2.36 | 0.1274 | 0.06 | 0.8137 |
| 1 × 3 | 14.95 | 0.0002 | 7.29 | 0.0081 | 0.81 | 0.3691 | 0.13 | 0.7201 |
| 2 × 3 | 0.04 | 0.8482 | 0.97 | 0.3271 | 0.82 | 0.3659 | 0.16 | 0.6915 |
| 1 × 4 | 1.56 | 0.2152 | 11.52 | 0.0010 | 18.91 | 0.0000 | 5.11 | 0.0259 |
| 2 × 4 | 0.91 | 0.3419 | 4.16 | 0.0441 | 0.53 | 0.4667 | 0.12 | 0.7282 |
| 3 × 4 | 5.28 | 0.0236 | 11.77 | 0.0009 | 0.05 | 0.8235 | 4.86 | 0.0297 |
| 1 × 2 × 3 | 2.42 | 0.1228 | 0.21 | 0.6463 | 2.02 | 0.1587 | 0.04 | 0.8333 |
| 1 × 2 × 4 | 0.00 | 0.9884 | 0.24 | 0.6235 | 0.11 | 0.7371 | 0.73 | 0.3960 |
| 1 × 3 × 4 | 4.89 | 0.0293 | 5.30 | 0.0233 | 0.03 | 0.8587 | 0.53 | 0.4677 |
| 2 × 3 × 4 | 0.01 | 0.9285 | 1.56 | 0.2152 | 0.00 | 0.9693 | 0.00 | 0.9960 |
| 1 × 2 × 3 × 4 | 0.06 | 0.8082 | 0.83 | 0.3639 | 0.65 | 0.4227 | 0.24 | 0.6243 |
|  | Mycorrhizal colonization | | MGR | | MPR | | MNR | |
|  | F | p | F | p | F | p | F | p |
| Plant (1) | 12.15 | 0.0010 | 123.10 | 0.0000 | 6.86 | 0.0116 | 62.85 | 0.0000 |
| Soil (2) | 0.00 | 0.9845 | 16.14 | 0.0002 | 0.21 | 0.6515 | 19.68 | 0.0000 |
| Phosphorus (3) | 49.04 | 0.0000 | 53.27 | 0.0000 | 77.66 | 0.0000 | 38.53 | 0.0000 |
| 1 × 2 | 0.09 | 0.7626 | 48.26 | 0.0000 | 11.95 | 0.0011 | 21.17 | 0.0000 |
| 1 × 3 | 1.09 | 0.3008 | 19.91 | 0.0000 | 0.02 | 0.8904 | 10.14 | 0.0025 |
| 2 × 3 | 3.25 | 0.0773 | 32.34 | 0.0000 | 7.42 | 0.0088 | 21.69 | 0.0000 |
| 1 × 2 × 3 | 0.83 | 0.3664 | 29.64 | 0.0000 | 9.34 | 0.0036 | 15.38 | 0.0003 |
